# Partial volume correction for arterial spin labeling using the inherent perfusion information of multiple measurements

**DOI:** 10.1186/s12938-019-0631-8

**Published:** 2019-02-04

**Authors:** Yang Liu, Ze Wang, Ruihua Liang, Zhengrong Liang, Hongbing Lu

**Affiliations:** 1 School of Biomedical Engineering, Fourth Military Medical University, Xi’an, China; 2 Department of Radiology, Temple University, Philadelphia, USA; 3 Department of Radiology, State University of New York at Stony Brook, Stony Brook, USA

**Keywords:** Partial volume (PV) effect, Arterial spin labeling (ASL), Expectation maximization (EM), Cerebral blood flow (CBF), Perfusion

## Abstract

**Background:**

Arterial spin labeling (ASL) provides a noninvasive way to measure cerebral blood flow (CBF). The CBF estimation from ASL is heavily contaminated by noise and the partial volume (PV) effect. The multiple measurements of perfusion signals in the ASL sequence are generally acquired and were averaged to suppress the noise. To correct the PV effect, several methods were proposed, but they were all performed directly on the averaged image, thereby ignoring the inherent perfusion information of mixed tissues that are embedded in multiple measurements. The aim of the present study is to correct the PV effect of ASL sequence using the inherent perfusion information in the multiple measurements.

**Methods:**

In this study, we first proposed a statistical perfusion model of mixed tissues based on the distribution of multiple measurements. Based on the tissue mixture that was obtained from the high-resolution structural image, a structure-based expectation maximization (sEM) scheme was developed to estimate the perfusion contributions of different tissues in a mixed voxel from its multiple measurements. Finally, the performance of the proposed method was evaluated using both computer simulations and in vivo data.

**Results:**

Compared to the widely used linear regression (LR) method, the proposed sEM-based method performs better on edge preservation, noise suppression, and lesion detection, and demonstrates a potential to estimate the CBF within a shorter scanning time. For in vivo data, the corrected CBF values of gray matter (GM) were independent of the GM probability, thereby indicating the effectiveness of the sEM-based method for the PV correction of the ASL sequence.

**Conclusions:**

This study validates the proposed sEM scheme for the statistical perfusion model of mixed tissues and demonstrates the effectiveness of using inherent perfusion information in the multiple measurements for PV correction of the ASL sequence.

## Background

The arterial spin labeling (ASL) sequence provides a noninvasive way to measure the cerebral blood flow (CBF) by utilizing the magnetically labeled arterial blood water as an endogenous tracer to create a “label” image [1] and subsequently repeats the process to create a “control” image without labeling the arterial blood. The subtraction of the label and control images becomes the perfusion image, which reflects the amount of the arterial blood that is delivered to each voxel after the transit time [2]. Due to fast scan of the label and control images, the perfusion image (label/control difference) is very noisy; therefore, multiple label/control image pairs are commonly acquired and are averaged to impress the noise.

The spatial resolution of the ASL sequence was approximately 3–6 mm. The CBF estimation was contaminated by the partial volume (PV) effect, which results in less accuracy of the CBF quantification [3]. For accurate PV correction, the perfusion contributions of different tissues inside a mixed voxel should be estimated separately [4]. Asllani et al. [5] proposed a linear regression (LR) method, in which the CBF values of both gray matter (GM) and white matter (WM) are assumed to be constant within an *n *× *n* × 1 regression kernel. Under this assumption, the LR method can generate the separate GM’s and WM’s CBF maps, but spatial smoothing may be also introduced into the CBF maps. Then, several methods have been proposed to alleviate the smoothing effect of the LR method [6–8]. For multiple inversion-time (TI) ASL data, Chappell et al. reported a PV correction method using a spatially regularized kinetic curve model [9]. To the best of our knowledge, all of the current PV correction methods were performed directly on the averaged image of the multiple label/control pairs, thereby ignoring the inherent perfusion information of the mixed tissues that are embedded in the multiple measurements.

The aim of the present study is to correct the PV effect of the ASL sequence by using the inherent perfusion information of multiple measurements. It was reported that the multiple measurements of the ASL sequence could be regarded as noisy realizations of the original distribution [10]. Therefore, for each voxel composed of mixed tissues, the PV correction problem turns to how to estimate the perfusion contributions of different tissues from multiple noisy measurements. Generally, for magnetic resonance imaging (MRI), the Rician noise model is widely accepted [11]. However, after the label/control difference operation, Gaussian noise is generally considered in the perfusion images of ASL sequence [12, 13]. For the purpose of this study, we first proposed a statistical perfusion model of mixed tissues for the ASL sequence, based on the Gaussian distribution of multiple measurements. With the tissue mixture information obtained from the high-resolution structural image, a structure-based expectation maximization (sEM) scheme was developed to estimate the perfusion contributions of the mixed tissues from multiple measurements.

## Methods

### Perfusion model of a mixed voxel

Considering the low spatial resolution, the GM, WM, and cerebrospinal fluid (CSF) may all contribute to the label/control difference signal, ∆*M*. No ASL signal typically arises from CSF [14]; therefore, the perfusion signal ∆*M* at the spatial position *i* can be described as

1 $$\Delta M_{i} = P_{iGM} \Delta M_{iGM} + P_{iWM} \Delta M_{iWM}$$

where *P*_*iGM*_ and *P*_*iWM*_ are proportions of GM and WM in the voxel *i*, respectively. ∆*M*_*iGM*_ and ∆*M*_*iWM*_ are the difference magnetizations for GM and WM, respectively.

In the current CBF calculation method, the CBF *f* of a tissue type is obtained by

2 $$f_{tissue} = \left( {\frac{{\Delta M_{tissue} }}{{M_{0} }}} \right)F_{tissue}$$

where *F*_*tissue*_ is a tissue-specific parameter, and *M*_0_ represents the equilibrium brain tissue magnetization obtained from the M0 image. For a mixed voxel, its CBF comes independently from the GM part ($f_{GM}^{P}$) and the WM part ($f_{WM}^{P}$) and can be described as

3 $${\text{CBF}} = f_{GM}^{P} + f_{WM}^{P} = \frac{{P_{iGM} F_{GM} }}{{M_{i0} }}\Delta M_{iGM} + \frac{{P_{iWM} F_{WM} }}{{M_{i0} }}\Delta M_{iWM}$$

For ASL perfusion studies, *P*_*iGM*_ and *P*_*iWM*_ can usually be estimated from a high-resolution structural image (e.g., T1 weighted image) of the same subject, and *F*_*GM*_ and *F*_*WM*_ can be derived from the two-compartment model for the ASL data [15]. Therefore, for a CBF estimation of a mixed voxel, the key problem is to estimate the magnetizations of GM and WM (i.e., ∆*M*_*iGM*_ and ∆*M*_*iWM*_) from multiple measurements.

### Statistical perfusion model of mixed tissues

As described in the Introduction section, multiple measurements could be regarded as noisy realizations of the original distribution [10], and Gaussian noise is generally considered in each measurement of the ASL sequence [12, 13]. Based on the Gaussian distribution of multiple ASL measurements, we first established a statistical perfusion model of mixed tissues.

#### 1. The statistical model of multiple measurements

In the spatial domain, index *i* is defined to represent the spatial position of a concerned voxel. The intensities of this voxel were acquired by multiple measurements that constitute a column vector **Y **= {*Y*_*it*_, *t *= 1, …,*T*}, where *T* is the number of multiple measurements collected. Based on the Gaussian assumption, each *Y*_*it*_ is a noisy observation of a random variable with a mean of $\bar{Y}_{i}$ and a variance of $\sigma_{i}^{2}$, i.e.,

4 $$Y_{it} = \bar{Y}_{i} + n$$

where *n* represents statistically independent noise in *Y*_*it*_ [16]. Since each measurement of the ASL sequence is independently scanned, the conditional probability of the measurement vector **Y** can be described as

5 $$p\left( {{\mathbf{Y}}\left| {\{ \bar{Y}_{i} \} ,\{ \sigma_{i}^{2} \} } \right.} \right) = \prod\limits_{t = 1}^{T} {p\left( {Y_{it} \left| {\bar{Y}} \right.,\sigma_{i}^{2} } \right)}$$

#### 2. Statistical perfusion model of mixed tissues

The observation *Y*_*it*_ contains perfusion contributions from GM and WM. The GM component is denoted by *X*_*itGM*_, with a mean of $\bar{X}_{iGM}$ and a variance of $\sigma_{iGM}^{2}$. The WM component is denoted by *X*_*itWM*_ with a mean of $\bar{X}_{iWM}$ and a variance of $\sigma_{iWM}^{2}$. Thus, we have

6 $$p\left( {{\mathbf{X}}\left| {\bar{X}_{iGM} ,\bar{X}_{iWM} ,\sigma_{iGM}^{2} ,\sigma_{iWM}^{2} } \right.} \right) = \prod\limits_{t = 1}^{T} {\left\{ {p\left( {X_{itGM} \left| {\bar{X}_{iGM} ,\sigma_{iGM}^{2} } \right.} \right)p\left( {X_{itWM} \left| {\bar{X}_{iWM} ,\sigma_{iWM}^{2} } \right.} \right)} \right\}}$$

where **X **= {*X*_*itGM*_ and *X*_*itWM*_, *t *= 1, …,*T*} represents a vector of size 2 × *T*, at position *i*.

The mean and variance values of each voxel can be calculated by the summation of all contributions at this voxel, i.e.,

7 $$\bar{Y}_{i} = \bar{X}_{iGM} + \bar{X}_{iWM} \;{\text{and}}\;\sigma_{i}^{2} = \sigma_{iGM}^{2} + \sigma_{iWM}^{2}$$

By combining the voxel-wise perfusion model in Eq. 3 with the above observation model, we have

8 $$\bar{X}_{iGM} = P_{iGM} \Delta M_{iGM} \;{\text{and}}\;\bar{X}_{iWM} = P_{iWM} \Delta M_{iWM}$$

9 $$\sigma_{iGM}^{2} = P_{iGM} S_{iGM} \;{\text{and}}\;\sigma_{iWM}^{2} = P_{iWM} S_{iWM}$$

where *S*_*iGM*_ and *S*_*iWM*_ represent the variance of the GM and the WM signal, respectively. In this study, the *P*_*iGM*_ and *P*_*iWM*_, which represent the proportions of GM and WM inside the concerned voxel *i*, can be estimated from the registered high-resolution structural image, which can be regarded as constants for a concerned voxel.

#### 3. Normal statistical model

For the ASL sequence, the perfusion signal contains GM and WM components. Suppose that each tissue type is independent and follows a Gaussian distribution. Equation 6 becomes

10 $$\begin{aligned} & p({\mathbf{X}}\left| {\Delta M_{iGM} ,\Delta M_{iWM} ,S_{iGM} ,S_{iWM} } \right.) \\ & = \prod\limits_{t = 1}^{T} {\left\{ {\left( {\frac{1}{{\sqrt {2\pi P_{iGM} S_{iGM} } }}e^{{ - \frac{{\left( {X_{itGM} - P_{iGM} \Delta M_{iGM} } \right)^{2} }}{{2P_{iGM} S_{iGM} }}}} } \right) \times \left( {\frac{1}{{\sqrt {2\pi P_{iWM} S_{iWM} } }}e^{{ - \frac{{\left( {X_{itWM} - P_{iWM} \Delta M_{iWM} } \right)^{2} }}{{2P_{iWM} S_{iWM} }}}} } \right)} \right\}} \\ \end{aligned}$$

The estimation of $p\left( {{\mathbf{Y}} |\Delta M_{iGM} ,\Delta M_{iWM} ,S_{iGM} ,S_{iWM} } \right)$ derived from Eq. 5 would generate several nonlinear equations, which are difficult to solve. Given $\bar{Y}_{i} = \bar{X}_{iGM} + \bar{X}_{iWM}$ in Eq. 7 and the description in Eq. 10, the EM algorithm may provide an alternative method and effective solution to estimate the model parameters {∆*M*_*iGM*_, ∆*M*_*iWM*_, *S*_*iGM*_, *S*_*iWM*_} based on the structural mixture information derived from a high-resolution image.

### EM algorithm for parameter estimation

In the EM approach [17, 18], the observation *Y*_*it*_ is regarded as an incomplete random variable. The *X*_*itGM*_ and *X*_*itWM*_ are regarded as complete variables, which can reflect the complete perfusion information at each measurement point *t* for a concerned voxel of position *i*. The probability distribution of the incomplete data {*Y*_*it*_} can be depicted by the complete data, {*X*_*itGM*_} and {*X*_*itWM*_}, using an integral equation under the condition of {*Y*_*it*_ = *X*_*itGM*_ + *X*_*itWM*_}:

11 $$\begin{aligned} & p\left( {Y_{it} \left| {\Delta M_{iGM} ,\Delta M_{iWM} ,S_{iGM} ,S_{iWM} } \right.} \right) \\ {\kern 1pt} & = \int_{{\left\{ {Y_{it} = X_{itGM} + X_{itWM} } \right\}}} {\left\{ {p\left( {X_{itGM} \left| {\bar{X}_{iGM} ,\sigma_{iGM}^{2} } \right.} \right)p\left( {X_{itWM} \left| {\bar{X}_{iWM} ,\sigma_{iWM}^{2} } \right.} \right)} \right\}dX} \\ \end{aligned}$$

In this study, the EM algorithm was used to seek a solution to maximize the conditional expectation of the complete data in Eq. 10. The *E*-*step* is to compute the conditional expectation. The *M*-*step* subsequently attempts to maximize the expectation of the complete-data log likelihood using the latent variables that were computed in the *E*-*step*, given the observations.

*E*-*step* This step computes the likelihood *p*(**X|**Θ) of the complete data in Eq. 10, given {*Y*_*it*_} and parameter $\varTheta^{(n)} = \left\{ {\Delta M_{iGM}^{(n)} ,\Delta M_{iWM}^{(n)} ,S_{iGM}^{(n)} ,S_{iWM}^{(n)} } \right\}$. The conditional expectation is depicted in Eq. 12.

12 $$\begin{aligned} Q(\varTheta |\varTheta^{(n)} ) = E_{{Y_{it} = X_{itGM} + X_{itWM} }} [\ln (p({\text{X}}|\varTheta ))|{\text{Y}},\varTheta^{(n)} ] = E_{{_{{Y_{it} = X_{itGM} + X_{itWM} }} }} \left[ { - \frac{1}{2}\sum\limits_{t} {\left\{ {\ln \left( {2\pi P_{iGM} S_{iGM} } \right) + \frac{1}{{P_{iGM} S_{iGM} }}\left[ {X_{itGM}^{2} - 2P_{iGM} \Delta M_{iGM} X_{itGM} + (P_{iGM} \Delta M_{iGM} )^{2} } \right]} \right\}} |Y_{it} ,\varTheta^{(n)} } \right] + E_{{_{{Y_{it} = X_{itGM} + X_{itWM} }} }} \left[ { - \frac{1}{2}\sum\limits_{t} {\left\{ {\ln \left( {2\pi P_{iWM} S_{iWM} } \right) + \frac{1}{{P_{iWM} S_{iWM} }}\left[ {X_{itWM}^{2} - 2P_{iWM} \Delta M_{iWM} X_{itWM} + (P_{iWM} \Delta M_{iWM} )^{2} } \right]} \right\}} |Y_{it} ,\varTheta^{(n)} } \right] = - \frac{1}{2}\sum\limits_{t} {\left\{ \begin{aligned} \ln \left( {2\pi P_{iGM} S_{iGM} } \right) + \frac{1}{{P_{iGM} S_{iGM} }}\left[ {E_{{Y_{it} = X_{itGM} + X_{itWM} }} (X_{itGM}^{2} |Y_{it} ,\varTheta^{(n)} ) - 2P_{iGM} \Delta M_{iGM} E_{{Y_{it} = X_{itGM} + X_{itWM} }} (X_{itGM} |Y_{it} ,\varTheta^{(n)} ) + (P_{iGM} \Delta M_{iGM} )^{2} } \right] + \hfill \\ \ln (2\pi P_{iWM} S_{iWM} ) + \frac{1}{{P_{iWM} S_{iWM} }}\left[ {E_{{_{{Y_{it} = X_{itGM} + X_{itWM} }} }} (X_{itWM}^{2} |Y_{it} ,\varTheta^{(n)} ) - 2P_{iWM} \Delta M_{iWM} E_{{Y_{it} = X_{itGM} + X_{itWM} }} (X_{itWM} |Y_{it} ,\varTheta^{(n)} ) + (P_{iWM} \Delta M_{iWM} )^{2} } \right] \hfill \\ \end{aligned} \right\}} \end{aligned}$$

Based on the deduction of the preceding conditional expectation, we have

13 $$\begin{aligned} X_{itGM}^{(n)} & = E_{{Y_{it} = X_{itGM} + X_{itWM} }} (X_{itGMt} |Y_{it} ,\varTheta^{(n)} ) \\ & = P_{iGM} \Delta M_{iGM}^{(n)} + \frac{{P_{iGM} S_{iGM}^{(n)} }}{{P_{iGM} S_{iGM}^{(n)} + P_{iWM} S_{iWM}^{(n)} }} \\ & \quad\quad \times \left[ {Y_{it} - (P_{iGM} \Delta M_{iGM}^{(n)} + P_{iWM} \Delta M_{iWM}^{(n)} )} \right] \\ \end{aligned}$$

14 $$\begin{aligned} X_{itWM}^{(n)} & = E_{{Y_{it} = X_{itGM} + X_{itWM} }} (X_{itWM}^{{}} |Y_{it} ,\varTheta^{(n)} ) \\ & = P_{iWM} \Delta M_{iWM}^{(n)} + \frac{{P_{iWM} S_{iWM}^{(n)} }}{{P_{iGM} S_{iGM}^{(n)} + P_{iWM} S_{iWM}^{(n)} }} \\ & \quad\quad \times {\kern 1pt} \left[ {Y_{it} - (P_{iGM} \Delta M_{iGM}^{(n)} + P_{iWM} \Delta M_{iWM}^{(n)} )} \right] \\ \end{aligned}$$

15 $$\begin{aligned}(X_{itGM}^{2} )^{(n)} &= E_{{_{{Y_{it} = X_{itGM} + X_{itWM} }} }} [X_{itGMt}^{2} |Y_{it} ,\varTheta^{(n)} ]{\kern 1pt} \\ & = (X_{itGM}^{(n)} )^{2} + \frac{{(P_{iGM} S_{iGM}^{(n)} )\left( {P_{iWM} S_{iWM}^{(n)} } \right)}}{{P_{iGM} S_{iGM}^{(n)} + P_{iWM} S_{iWM}^{(n)} }} \end{aligned}$$

16 $$\begin{aligned} (X_{itWM}^{2} )^{(n)} &= E_{{Y_{it} = X_{itGM} + X_{itWM} }} [X_{itWM}^{2} |Y_{it} ,\varTheta^{(n)} ]{\kern 1pt} \\ &= (X_{itWM}^{(n)} )^{2} + \frac{{(P_{iGM} S_{iGM}^{(n)} )(P_{iWM} S_{iWM}^{(n)} )}}{{P_{iGM} S_{iGM}^{(n)} + P_{iWM} S_{iWM}^{(n)} }} \end{aligned}$$

*M*-*step*: This step maximizes the conditional expectation to estimate the next iteration $\left\{ {\Delta M_{iGM}^{{(n{ + }1)}} ,\Delta M_{iWM}^{{(n{ + }1)}} ,S_{iGM}^{{(n{ + }1)}} ,S_{iWM}^{{(n{ + }1)}} } \right\}$, which can be described as

17 $$\frac{\partial Q}{{\partial \Delta M_{iGM} }}|_{{\Delta M_{iGM} = \Delta M_{iGM}^{(n + 1)} }} = 0 \Rightarrow \Delta M_{iGM}^{(n + 1)} = \frac{{\sum\nolimits_{t = 1}^{T} {X_{itGM}^{(n)} } }}{{T \cdot P_{iGM} }}$$

18 $$\frac{\partial Q}{{\partial \Delta M_{iWM} }}|_{{\Delta M_{iWM} = \Delta M_{iWM}^{(n + 1)} }} = 0 \Rightarrow \Delta M_{iWM}^{(n + 1)} = \frac{{\sum\nolimits_{t = 1}^{T} {X_{itWM}^{(n)} } }}{{T \cdot P_{iWM} }}$$

19 $$S_{iGM}^{(n + 1)} = \frac{{\sum\nolimits_{t = 1}^{T} {\left[ {(X_{itGM}^{2} )^{(n)} - 2X_{itGM}^{(n)} P_{iGM} \Delta M_{iGM}^{(n)} + (P_{iGM} \Delta M_{iGM}^{(n)} )^{2} } \right]} }}{{T \cdot P_{iGM} }}$$

20 $$S_{iWM}^{(n + 1)} = \frac{{\sum\nolimits_{t = 1}^{T} {\left[ {(X_{itWM}^{2} )^{(n)} - 2X_{itWM}^{(n)} P_{iWM} \Delta M_{iWM}^{(n)} + (P_{iWM} \Delta M_{iWM}^{(n)} )^{2} } \right]} }}{{T \cdot P_{iWM} }}$$

Based on the proposed sEM algorithm, we can estimate ∆*M*_*iGM*_ and ∆*M*_*iWM*_ using the multiple measurements of the ASL sequence.

### Implementation of the sEM scheme for PV correction

The implementation of the proposed sEM scheme for PV correction can be summarized as follows:

1. Segmentation of high-resolution structural image. The segmented results and ASL data are co-registered. For each mixed voxel at position *i*, the percentages of GM and WM, *P*_*iGM*_ and *P*_*iWM*_, were obtained.
2. Initialization of the model parameters $\left\{ {\Delta M_{iGM}^{(0)} ,S_{iGM}^{(0)} ,\Delta M_{iWM}^{(0)} ,S_{iWM}^{(0)} } \right\}$.
3. Constitute a column vector with all measurements of the mixed voxel at position *i*.
4. Iterative estimation of GM and WM components for the mixed voxel at position *i* using the column vector in step (3), following Eqs. 17–20.
5. Repeat steps (3) and (4) for the next voxel until all the voxels are corrected.

## Material and evaluation

In this study, the performance of the proposed sEM scheme was evaluated by both digital simulations and clinical data. The two simulations listed below were designed to evaluate its performance quantitatively, especially with regards to noise reduction, lesion detection, and its potential to estimate CBF from fewer measurements. After the simulation studies, the in vivo ASL data were used to evaluate the clinical feasibility.

### Simulation 1

In this simulation, a digital head phantom was generated from a structural MRI brain dataset with a voxel size of 1 × 1 × 1 mm^3^. After the normalization and segmentation of the MRI data using SPM8 software, the posterior probability images of GM and WM were generated. Next, the images were masked to remove the voxels with probabilities lower than 0.1 [7, 9]. The head phantom was simulated as follows:

1. The probability images were resampled to a size of 60 × 72 × 60, with a spatial resolution of 3 × 3 × 3 mm^3^ using SPM8.
2. Across the whole brain, the WM region was simulated as 20 mL/100 g/min.
3. The GM was simulated as 60 mL/100 g/min, with a hypo-perfused region (30 mL/100 g/min) and a hyper-perfused region (90 mL/100 g/min). Both of the regions were spherical regions with a radius of 5.
4. Based on the probability images and the signals of GM and WM, the perfusion signal of each voxel in the 3D perfusion image was generated according to Eq. 1.
5. It was reported that the noise level of the ASL data ranges from 6.7 to 13.2 according to different labeling schemes and readout sequences [19]. To evaluate the noise impact on PV correction, three different levels of Gaussian noise, with a standard deviation (std) of 5, 10 and 15, respectively, were added into the 3D perfusion image to generate low-, middle-, and high-noise realizations. The highest noise was approximately 25% (15/60) of the GM signal.
6. Generally, the number of label/control pairs is set as 40–60. To evaluate the proposed method, 40 noisy realizations were generated for each ASL sequence.

### Simulation 2

To evaluate the benefit of PV correction on the lesion detection of small CBF alterations, in this simulation, three regions with different sizes and simulated values, instead of the two regions used in step (3) of Simulation 1, were simulated inside the homogeneous GM tissues: (1) a spherical region of radius 5 with CBF of 75 mL/100 g/min, (2) a 3 × 3 × 3 cubic region with CBF of 45 mL/100 g/min, and (3) a 2 × 2 × 2 cubic region with CBF of 75 mL/100 g/min. The difference between the three regions and the homogeneous GM region were selected from the high std of noise, i.e., 15.

### In vivo data

To test the feasibility of PV correction on in vivo ASL data, the ASL scans were collected from three healthy subjects, which were acquired by a Siemens 3T scanner using the pseudo-continuous ASL perfusion imaging sequence with gradient-echo echoplanar imaging (EPI). The acquisition parameters were TR = 4 s, TE = 11 ms, FOV = 220 × 220 mm^2^, voxel size = 3.4 × 3.4 × 5 mm^3^, matrix = 64 × 64 × 20, flip angle = 90°, and post labeling delay = 1.5 s. Forty label/control pairs were acquired. A high-resolution structural image was also acquired with the following parameters: TR = 1900 ms, TE = 2.9 ms, FOV = 250 × 250 mm^2^, matrix = 256 × 256 × 176, and flip angle = 90°.

The ASL and structural images were preprocessed using SPM8. For each subject, the ASL images was realigned separately for the label and control image series. After realignment, the images were normalized, followed by pair-wise subtraction. The corresponding structural image was normalized and segmented to generate probability images of GM and WM, which were later masked with probabilities lower than 0.1. Finally, the probability images were co-registered with ASL data to obtain *P*_*iGM*_ and *P*_*iWM*_ at each position *i*, using a transformation of the structural and ASL coordinates with an MNI coordinate.

### Comparison of PV correction

As is well-known, the EM algorithm is quite sensitive to the initialization. Considering the limited number of measurements and the intensive computation load of the EM algorithm, a relatively accurate initialization from an estimation that uses an uncorrected image or other spatial PV correction method (e.g., the LR method) would lead to accurate estimations and fast convergence. To compare the effect of the PV correction using different methods, the simulated data and the in vivo data were all analyzed using:

1. No correction. The averaged image was used as the result.
2. The LR method. The averaged image was used to separately estimate the GM and the WM CBF maps using the LR method with a 5 × 5 × 1 regression kernel, which was suggested to provide the best compromise between smoothing and PV correction [5, 7].
3. The sEM method, which is the EM algorithm initialized with an estimation from no correction. In this method, $\Delta M_{iGM}^{(0)}$ and $\Delta M_{iWM}^{(0)}$ were set as the mean value of GM and WM regions from no correction, and $S_{iGM}^{(0)}$ and $S_{iWM}^{(0)}$ were set as the std of GM and WM. The iteration number was set as 100 to ensure the convergence.
4. The sEM-LR method, which is the EM algorithm initialized with the LR method. In this method, each 3D difference image was first corrected with the LR method to obtain the initialization of $\left\{ {\Delta M_{iGM}^{(0)} ,\Delta M_{iWM}^{(0)} ,S_{iGM}^{(0)} ,S_{iWM}^{(0)} } \right\}$. With this initialization, the GM and WM maps were estimated using the sEM method. The iteration number was also set as 100 to ensure the convergence.

For the simulation data, the root mean square error (RMSE) analysis was performed for a quantitative evaluation of these correction methods.

For the in vivo data, the GM CBF ratio, which is the ratio between the estimated GM CBF and the mean GM CBF of the uncorrected maps, was calculated for each voxel. This index can avoid the bias introduced from a different calibration method in which the CBF value is calculated and permits the assessment of the relative CBF changes after correction [9].

### The region of interest (ROI) analysis

In this study, the consistency of the mean GM CBF across the whole range of GM probabilities was used to quantitatively evaluate the estimated results from different PV correction methods. To this aim, nine ROIs were automatically defined based on the GM probability images, with the probability range between [10–20%], [20–30%],…, [90–100%], respectively. Next, the mean value of GM CBF in each ROI was calculated. It should be noted that the less independent are the GM CBF values from the GM probability, the better the performance of PV correction is.

## Results

### Simulation results

**Fig. 1:**
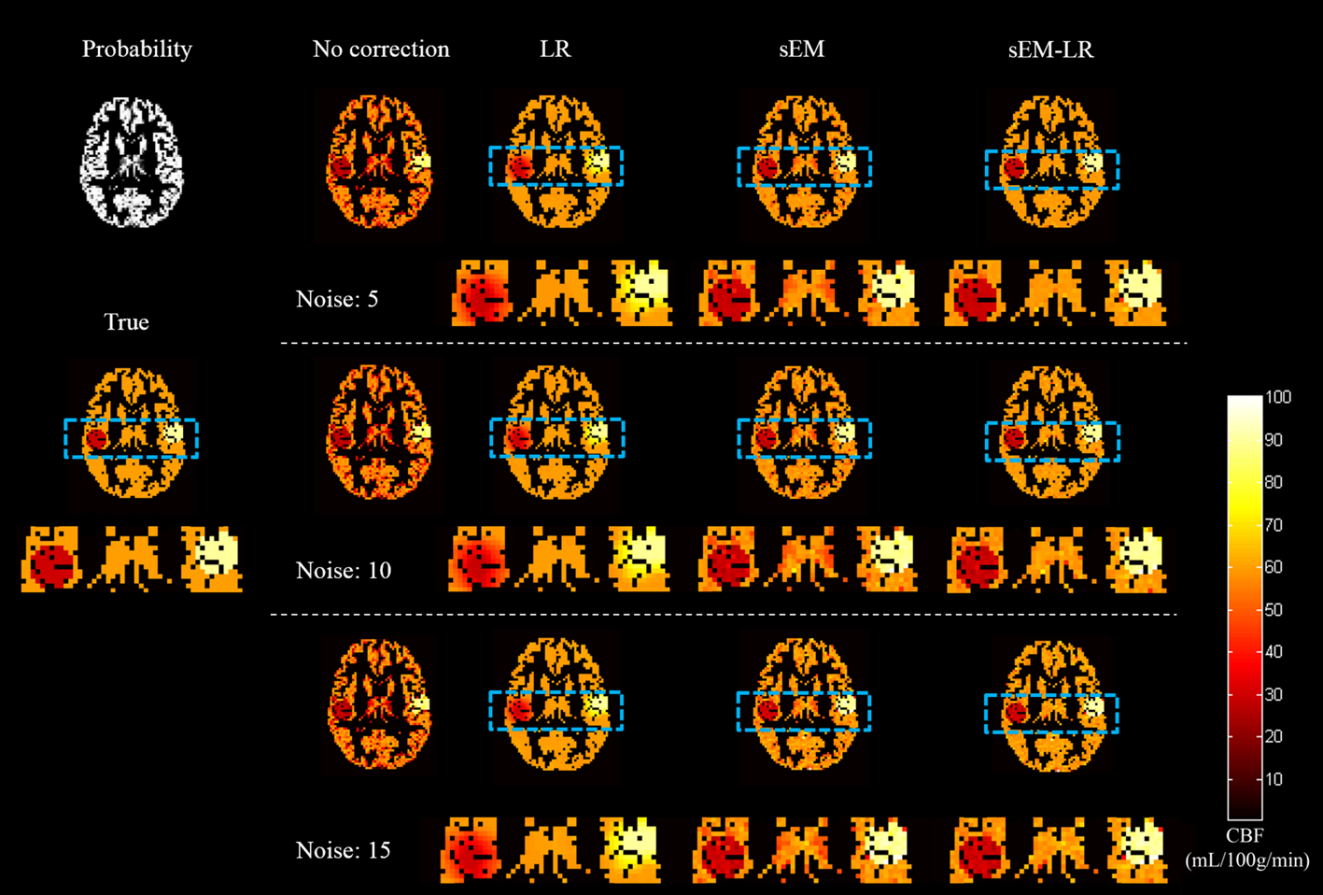
GM CBF maps (middle slice) estimated using different correction methods under different noise levels. From left to right: no correction, LR, sEM, and sEM-LR methods. From top to bottom: different levels of Gaussian noise, with a standard deviation of 5, 10 and 15, respectively. The dotted box areas of ground truth and the corrected results with LR, sEM and sEM-LR were magnified into view

Figure 1 shows the middle slice of the GM CBF estimation for Simulation 1 using no correction, LR, sEM, and sEM-LR methods. Clearly, the CBF maps derived from the LR, sEM, and sEM-LR methods outperformed those of no correction, with less noise and better restoration. At the edges of hypo- and hyper-CBF regions, the GM map that was estimated by the LR method exhibited a visible smoothing effect.

**Fig. 2:**
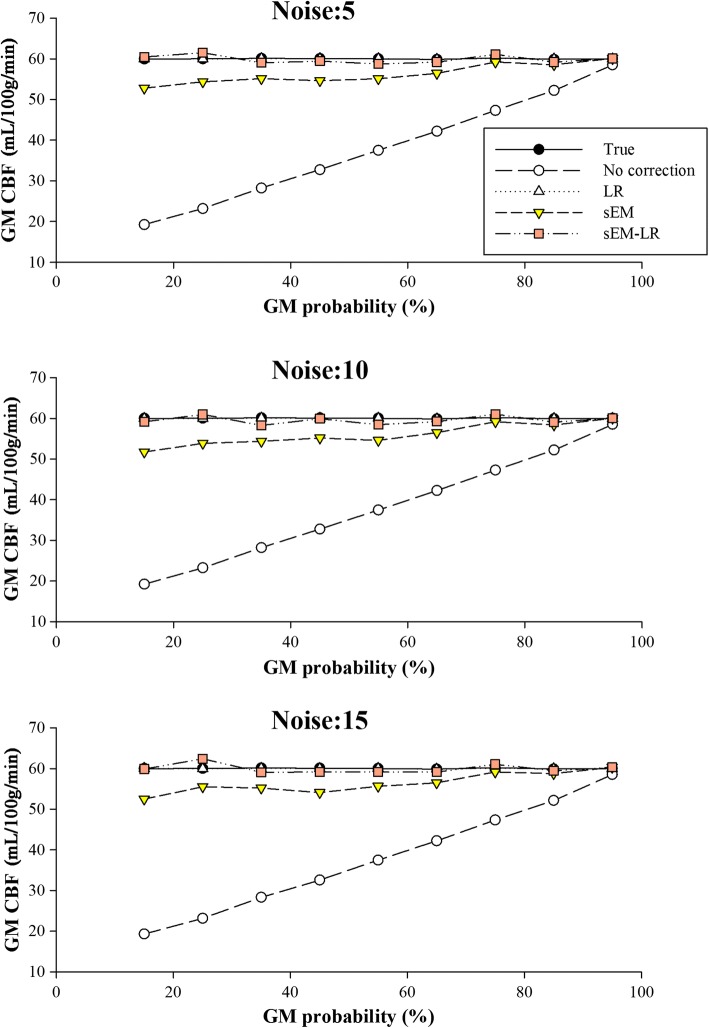
ROI analysis for GM CBF under different noise levels. Each data point represents the mean GM CBF for all voxels falling within a 10 percentile range of the GM probability. From top to bottom: different levels of Gaussian noise, with a standard deviation of 5, 10 and 15, respectively

Figure 2 shows the results of ROI analysis using Simulation 1 when the different PV-corrected methods were performed. It demonstrated that the GM CBF estimation using no correction was underestimated, compared with the ground truth. Corrected by the LR and sEM-LR methods, the GM CBF curves of different GM probabilities were almost consistent with the true line, while that of sEM method was a little underestimated at the relative low GM probability. The performance of the LR and the two sEM-based methods seems to be less affected by the noise level.

**Fig. 3:**
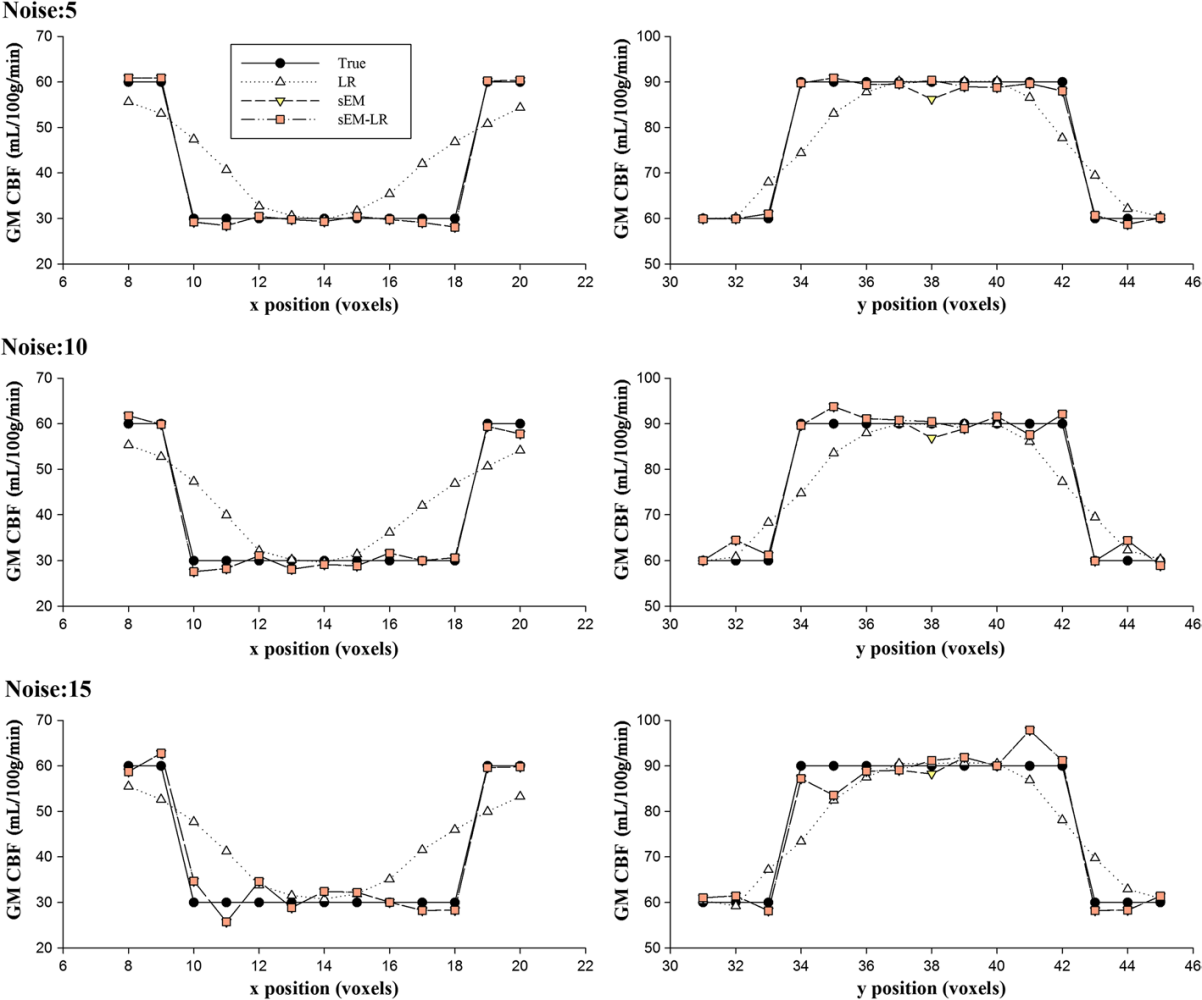
The profiles of the GM CBF estimation through the center of the hypo- and hyper-perfusion region in the slice shown in Fig. 1. From top to bottom: different levels of Gaussian noise, with a standard deviation of 5, 10 and 15, respectively

To illustrate the effect of different correction methods on the CBF accuracy under different noise levels, the profiles of the lines passing the centers of the hypo- and hyper-CBF regions of the GM CBF maps are shown in Fig. 3, which demonstrates that the sEM and sEM-LR methods provided accurate GM CBF estimations with preserved details and tissue interfaces but are affected by the noise level. Table 1 gives the RMSE values of the estimated CBF maps and the true map, and the differences between them indicated that the sEM-LR method outperformed the LR method at different noise levels.

**Table 1:** RMSE between the estimated GM CBF and true values in Simulation 1 using different methods (unit: mL/100 g/min)

	Noise: 5	Noise:10	Noise: 15
LR	3.5621	3.6076	3.6243
sEM	3.8028	4.2388	4.8365
sEM-LR	1.4603	2.4051	3.4356

**Fig. 4:**
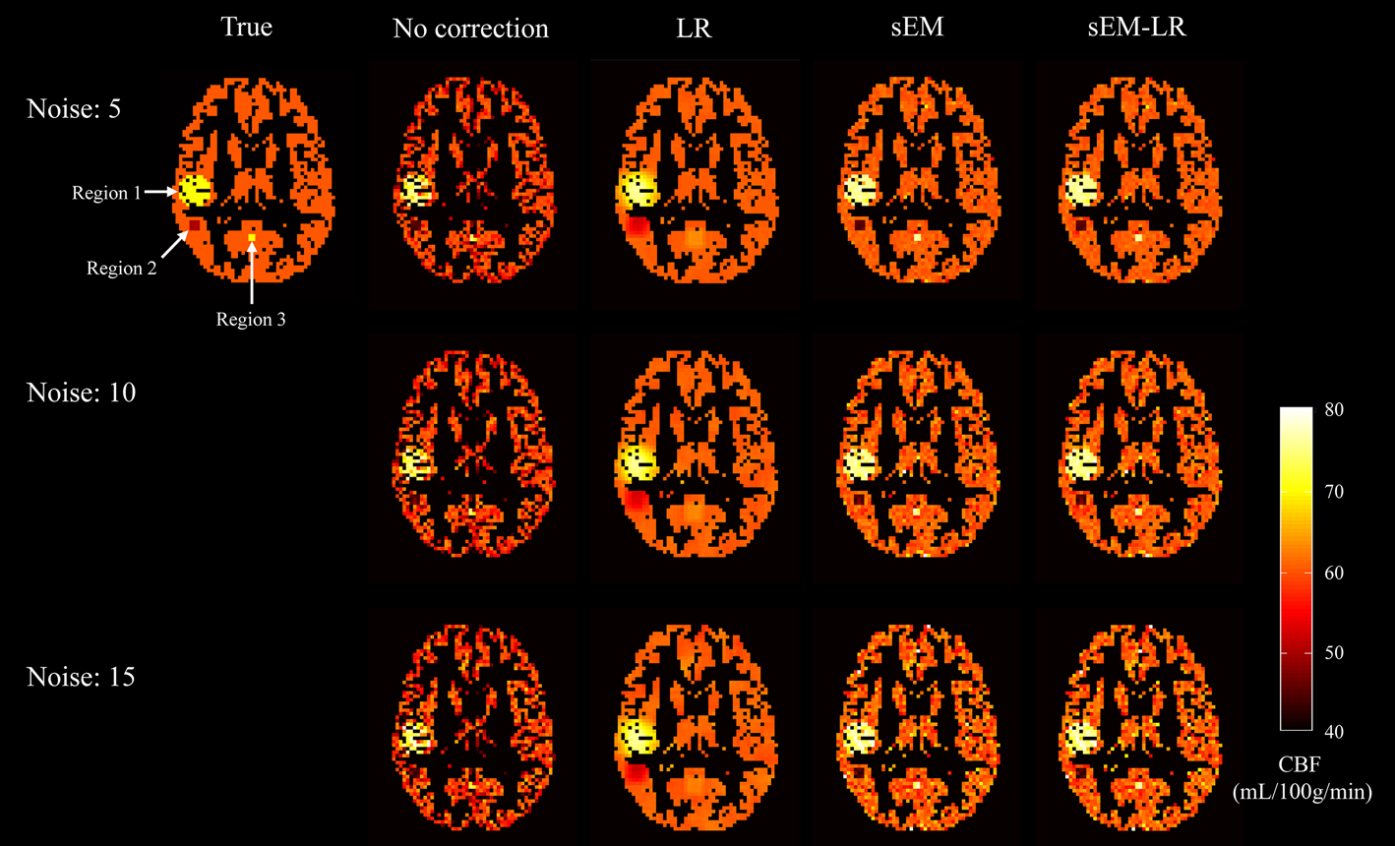
Detection of small lesions using different correction methods. Region 1: a spherical region of radius 5 with CBF of 75 mL/100 g/min, region 2: a 3 × 3 × 3 cubic region with CBF of 45 mL/100 g/min, region 3: a 2 × 2 × 2 cubic region with CBF of 75 mL/100 g/min. From left to right: no correction, LR, sEM, and sEM-LR methods. From top to bottom: different levels of Gaussian noise, with a standard deviation of 5, 10 and 15, respectively

The effect of the PV correction on lesion detection is shown in Fig. 4. It is obvious that, although the alterations were small, all of the regions with CBF alterations can be detected by using two sEM-based methods, even if the std of the noise was same as the CBF alteration. However, the two small regions (region 2 and region 3 in Fig. 4) were difficult to detect when corrected by the LR method.

**Fig. 5:**
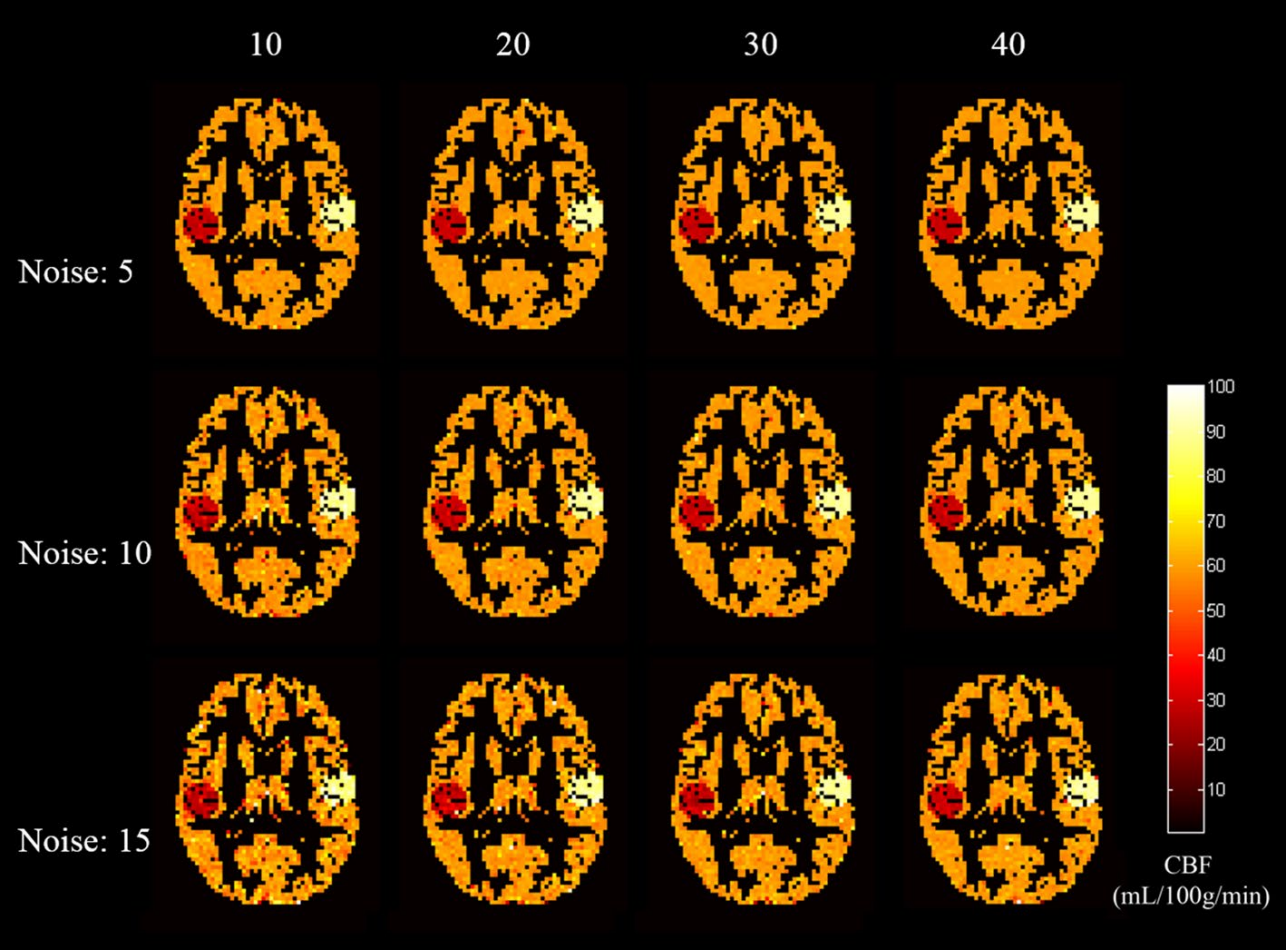
The CBF results estimated from different numbers of the label/control pairs using the sEM-LR method. From top to bottom: different levels of Gaussian noise, with a standard deviation of 5, 10 and 15, respectively

Figure 5 demonstrates the GM CBF maps (middle slice) that were estimated from fewer measurements, which indicate that with the increase of measurement numbers, the CBF estimation was more accurate and was less affected by noise. The RMSE values of the CBF maps that were estimated from different numbers of measurements are listed in Table 2, which also illustrate that the restoration was better with the increased number of multiple measurements. In most cases, the RMSEs using the sEM-LR method with fewer measurements (Table 2) were lower than those of the LR method with normal measurements (the corresponding RMSE shown in Table 1).

**Table 2:** RMSE between the estimated GM CBF and true values under different numbers of label/control pairs, when using the sEM-LR algorithm (unit: mL/100 g/min)

Std of noise	Number of label/control pairs			
	10	20	30	40
5	2.7288	2.0557	1.6624	1.4603
10	5.7988	3.2497	3.0240	2.4051
15	7.5917	5.4996	4.5987	3.4356

The computation times of each correction method to correct Simulation 1 were compared using the same computer (Intel CPU E3-1240, RAM of 16G). The computation time of the LR method for the 60 × 72 × 60 averaged image was 19.2 s. With the stopping criterion of 100 iterations, the computation costs for the sEM and sEM-LR methods were 177 s and 982 s, respectively. With the stopping rule of the difference between two adjacent iterations less than 0.001, the time costs of them were 4 s and 792 s, respectively. It should be noticed that the majority time of the sEM-LR was used for the initialization of all spatial label/control difference images using the LR method, which was about 790 s.

**Table 3:** The standard deviation of CBF ratio for three subjects, using different methods

	No correction	LR	sEM	sEM-LR
Subject 1	0.111	0.030	0.061	0.060
Subject 2	0.091	0.040	0.049	0.039
Subject 3	0.078	0.036	0.055	0.060

### In vivo data

**Fig. 6:**
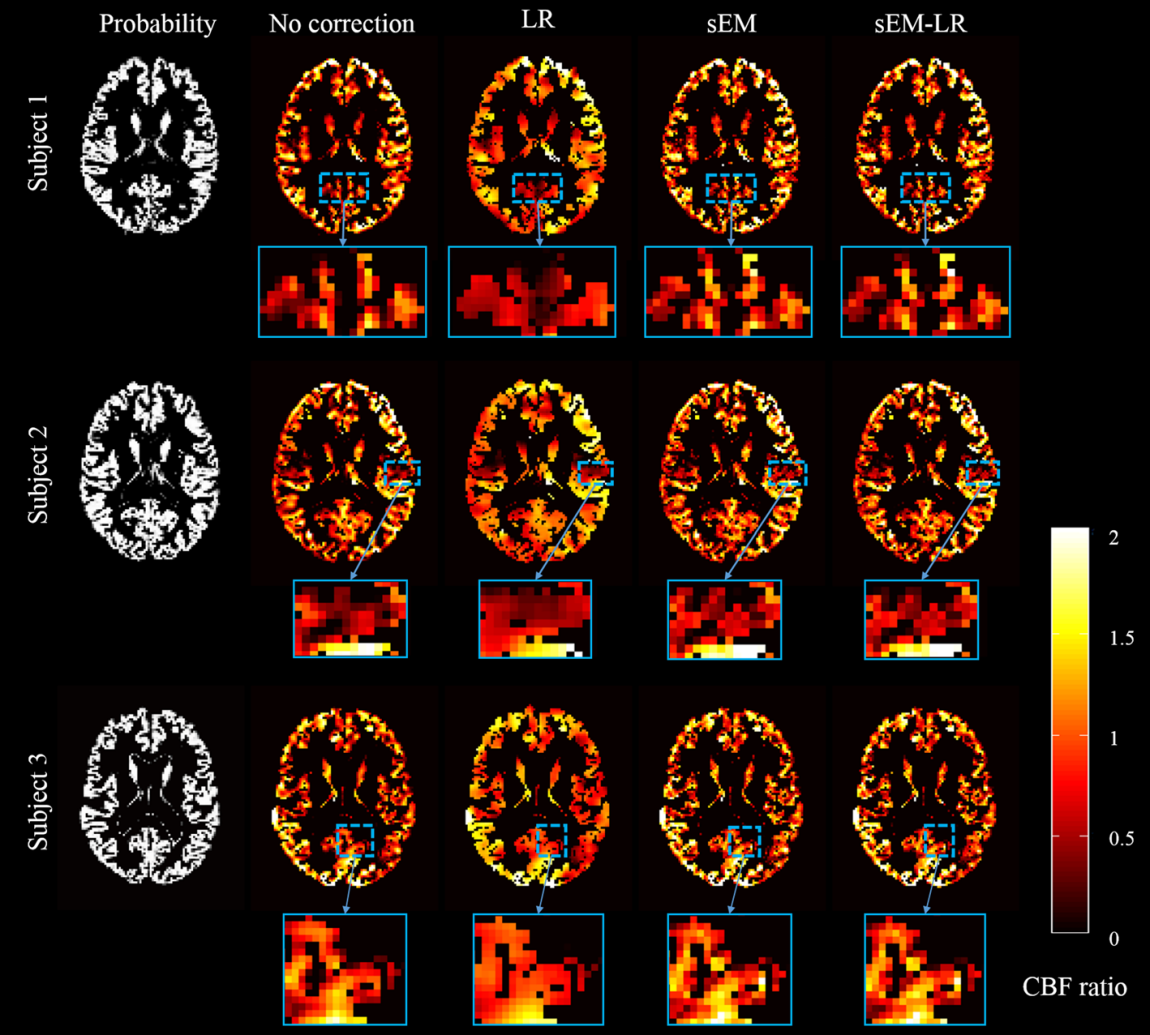
Estimated results (middle slice) from three healthy subjects, which show the GM CBF ratio (the estimated GM value to the mean GM CBF without PV correction). From left to right: probability, no correction, LR, sEM, and sEM-LR methods. The GM CBF images have been masked at a GM probability > 10%

Figure 6 gives the GM CBF ratio of three subjects by using different correction methods. For a better demonstration of the results, the regions enclosed within dotted boxes were zoomed. Compared with the results without correction and estimated from LR method, the proposed sEM and sEM-LR methods reserved more details, especially at the tissue interface.

**Fig. 7:**
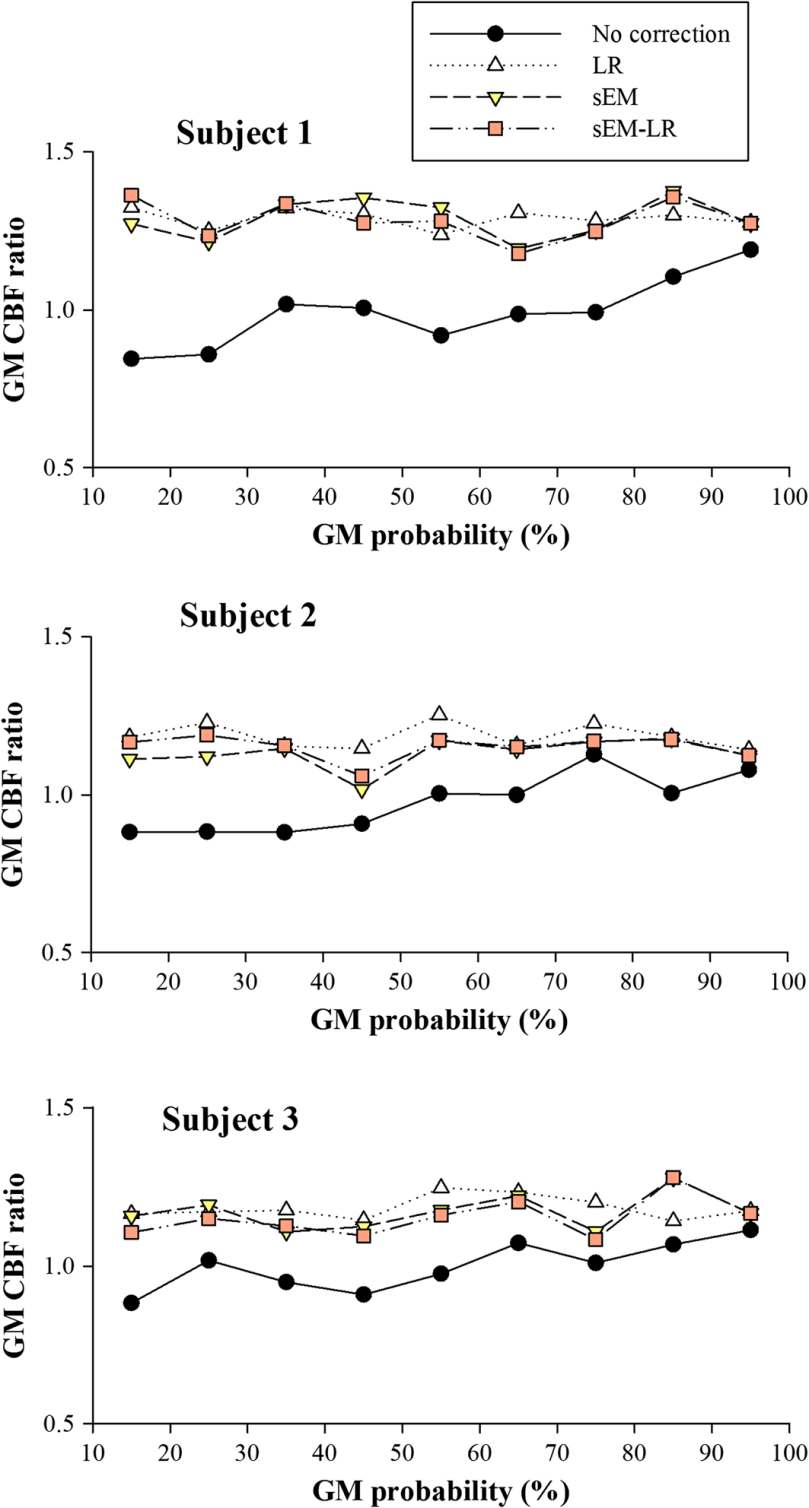
ROI analysis for three healthy subjects shown in Fig. 6; each data point represents the mean GM CBF for all voxels falling within a 10 percentile range of the GM probability. From top to bottom: each healthy subject for in vivo data

Figure 7 shows the ROI analysis of the ASL data using different methods. For each subject, the results of the LR and the two sEM-based methods demonstrate less variation (lower standard deviation) than that of the uncorrected data (Table 3), which indicate less independence of the GM CBF values from the GM probability.

## Discussion

The present study proposed a sEM scheme for the PV correction of the ASL sequence. For an accurate estimation of CBF, a statistical perfusion model of mixed tissues was first established. Then, based on the prior tissue mixture obtained from a high-resolution structural image, a structure-based EM algorithm (sEM scheme) was proposed to estimate the perfusion contributions of GM and WM tissues of the mixed voxels from multiple measurements of the ASL sequence. When the contributions of different tissues were estimated, the PV effect embedded in the multiple measurements was naturally resolved.

Different from the previous PV correction studies, the proposed method innovatively utilizes multiple measurements of label/control differences (perfusion images), instead of using the simple averaged image, to estimate the CBF contribution of the GM and WM components in each mixed voxel. The evaluation using computer simulations and the in vivo data demonstrated its superiority in PV correction, especially in the following aspects: (1) Edge preservation. Since the CBF contributions were estimated iteratively from the multiple measurements of a mixed pixel, with less influence from neighboring voxels, the EM estimation was superior in edge preservation and could detect small lesions with a radius of approximately 3.4 mm (calculated from a spherical volume of 2 × 2 × 2 m^3^ cube). (2) Noise suppression. Unlike the simple averaging of multiple noisy measurements, the sEM scheme restored the GM and WM components from a series of noisy realizations with Gaussian distribution. Thus, the scheme could not only suppress noise, but also could detect small CBF signals effectively, even if strong noise was applied. (3) Fast scan. The CBF estimation using fewer measurements indicated that the proposed method could achieve reasonable imaging quality with fewer label/control pairs and have the potential to shorten the scan time.

Unlike our previous work in which the EM algorithm was used to estimate the tissue mixture inside a mixed voxel [18, 20], in this study, we attempted to integrate the 3D structural image with perfusion series and develop a new sEM scheme for the perfusion estimation of different tissues in a mixed voxel from the multiple measurements of the ASL sequence. Since the contributions of GM and WM to the perfusion signal are independent and different, the proposed sEM scheme could estimate their different contributions effectively. However, if they are correlated or contribute same to the perfusion signal, the sEM method would not help, in which the simple averaging should be good enough.

It is known that the EM algorithm is quite sensitive to the initialization. If the initial values of the model parameters, such as ∆*M*_*iGM*_ and ∆*M*_*iWM*_, can be set as close as possible to the true values, better estimations could be obtained with fast convergence. To evaluate the effect of parameter initialization on the CBF estimation, the EM algorithm initialized with parameters estimated without correction and those estimated using the LR method were performed on both simulated and in vivo data. The results indicated that both sEM-based methods (sEM and sEM-LR) outperformed the LR method, while the sEM-LR method performed better than the sEM method only at relatively low GM probabilities (Fig. 2). Following the Markov random field model, the perfusion of a voxel is generally affected by neighboring voxels [21]. Since the proposed sEM method only considers perfusion correction from multiple measurements of the same voxel, a more accurate CBF estimation could be expected if spatial correction is considered further. Therefore, the combination of the proposed sEM with spatial prior obtained from the LR method, i.e., the sEM-LR method, could achieve better performance with the consideration of a spatial neighborhood.

Considering the iterative nature of the EM algorithm, the computation load of different methods was compared. The results indicated that the time cost of the sEM correction was comparable with other methods if a reasonable stopping criterion was used. The major cost of the sEM-LR method came from the initialization of all spatially different images by using the LR method, and not from EM optimization itself. The results also suggest that the use of the difference between two adjacent iterations that were less than 0.001 as the stopping criterion could reduce the computation time remarkably, because most voxels without the tissue mixture could reach the criterion very quickly. If parallel computation was performed, the computation time will be further greatly reduced.

Several limitations of this study should be addressed. Firstly, the proposed method needs multiple measurement information to correct PV effect, thus, this method is more suitable for the ASL sequence with time series, not for 3D ASL sequence. Secondly, the present study assumed that the voxels located at the same 3D spatial position differed only in noise. In practice, the distribution may be affected by temporal CBF variation, which may induce a bias of the CBF estimation for the in vivo data. In this study, we focus on the feasibility to use multiple measurements for an accurate CBF estimation under this assumption, and further studies will be performed to investigate the PV correction by using multiple measurements with consideration of temporal CBF variation. Although further improvement is required, this study validates the proposed statistical perfusion model and demonstrates the effectiveness and necessity of using inherent perfusion information in multiple measurements for PV correction of the ASL sequence.

## Conclusions

In this study, we proposed a statistical perfusion model of mixed tissues for each voxel of the ASL data. Based on this model, the sEM scheme was developed to estimate the contributions of different tissues to the perfusion signal of the mixed voxel with its multiple measurements. Compared to the traditional PV-corrected method, the proposed sEM-based method performs better in edge preservation, noise suppression, and lesion detection while demonstrating the potential to estimate CBF within a shorter scanning time. The results also indicated the effectiveness of using inherent perfusion information in multiple measurements for PV correction of the ASL sequence.

## Abbreviations

ASL: arterial spin labeling
CBF: cerebral blood flow
PV: partial volume
EM: expectation maximization
MRI: magnetic resonance imaging
LR: linear regression
